# Empowering aspects for healthy food and physical activity habits: adolescents’ experiences of a school-based intervention in a disadvantaged urban community

**DOI:** 10.1080/17482631.2018.1487759

**Published:** 2018-07-04

**Authors:** Christopher Holmberg, Christel Larsson, Peter Korp, Eva-Carin Lindgren, Linus Jonsson, Andreas Fröberg, John E Chaplin, Christina Berg

**Affiliations:** a Department of Food and Nutrition, and Sport Science, University of Gothenburg, Gothenburg, Sweden; b School of Health and Welfare, Halmstad University, Halmstad, Sweden; c Department of Pediatrics, Sahlgrenska Academy, University of Gothenburg and Sahlgrenska University Hospital, Gothenburg, Sweden

**Keywords:** Adolescence, empowerment, focus group interviews, food habits, health equity, health promotion, intervention, physical activity

## Abstract

**Purpose:**This study aimed to describe adolescents’ experiences of participating in a health-promoting school-based intervention regarding food and physical activity, with a focus on empowering aspects. **Method:** The school was located in a urban disadvantaged community in Sweden, characterized by poorer self-reported health and lower life expectancy than the municipality average. Focus group interviews with adolescents (29 girls, 20 boys, 14–15 years) and their teachers (n = 4) were conducted two years after intervention. Data were categorized using qualitative content analysis. **Results:** A theme was generated, intersecting with all the categories: Gaining control over one’s health: deciding, trying, and practicing together, in new ways, using reflective tools. The adolescents appreciated influencing the components of the intervention and collaborating with peers in active learning activities such as practicing sports and preparing meals. They also reported acquiring new health information, that trying new activities was inspiring, and the use of pedometers and photo-food diaries helped them reflect on their health behaviours. The adolescents’ experiences were also echoed by their teachers. **Conclusions:** To facilitate empowerment and stimulate learning, health-promotion interventions targeting adolescents could enable active learning activities in groups, by using visualizing tools to facilitate self-reflection, and allowing adolescents to influence intervention activities.

## Introduction

Social inequalities in health comprise a gradient throughout society with the greatest health problems found in vulnerable groups within society (the Swedish Commission for Equity in Health, 2017). To obtain more equity in health, it is important to address health-related behaviours (the Swedish Commission for Equity in Health, 2017). Adolescents’ socioeconomic status (SES) and their ethnicity have been identified as contributing factors for unhealthy eating (Mattisson, 2016) and physical activity (Stalsberg & Pedersen, 2010). These health habits have the potential to decrease the risk of illness and can thus contribute to reducing the health gap between adolescents from different SES groups (Mattisson, 2016; World Health Organization, 2016).

The evidence for the long-term health benefits of physical activity and for a diet rich in vegetables, legumes, and whole grains are compelling (Lee et al., 2012; Onvani, Haghighatdoost, Surkan, Larijani, & Azadbakht, 2016). The primary research priority might be to find ways in which to successfully support adolescents to develop and maintain sustainable healthy lifestyle habits. To engage in health-promotion interventions, adolescents need to feel that the health activities are relevant for them (Greene, 2013). However, many interventions have used predetermined designs based on the researchers’ perspectives with less focus on the adolescents’ own thoughts about what they perceive as meaningful for their health (World Health Organization, 2009).

### Empowerment as a means and an outcome of interventions

To facilitate adolescents’ active engagement in their own health, one needs to acknowledge the power structures that are inherent in all relationships pertinent to human health (Larsson & Jormfeldt, 2017), particularly when the adolescents belong to a disadvantaged group and lack information about available health resources due to, for example, being new in the country. Few health-promotion interventions have focused on adolescents from low-SES groups or from ethnic minority populations (Van Cauwenberghe et al., 2010). Less is known about such adolescents’ health-related experiences, so it is important to be open about their perceived health needs.

Openess and particpation is thus a key aspect of empowerment-based interventions. Interventions rely on active participation and participants that have a shared interest in the topic and cause are generally more active and engaged (Laverack, 2017). Adolescents can be seen as empowered rather than exploited when one listens to what they have to say, and whether they want to be included at all, by feeling that the issue is important and significant for them (Allard, 1996). Thus, a too strict focus on behaviour change might overlook the individuals own perceptions of what is important, which also increase the risk of unsuccessful interventions (Tengland, 2012).

Health-promoting interventions that intends to be empowering allows the participants to influence the programme, by for example setting the agenda or releasing certain resources. The added benefit of empowerment is that it provides the individuals with greater control in attaining healthy lifestyles that are sustainable (Laverack, 2017). According to Shier (2001), participation includes such aspects as listening to the adolescents, supporting them in expressing their views, taking the adolescents’ views into consideration, including them in decision-making processes, and sharing responsibility for decision-making with the adolescents. Participation is often conceptualized as both a process and a valued outcome in health-promotion programs (Potvin, 2007). Jacquez, Vaughn, and Wagner (2013) conducted a literature review including studies that had utilized participatory research, finding that most studies were cross-sectional in nature and that the researchers partnered with adolescents in only half of the studies. Many intervention studies targeting adolescents have focused mainly on obesity prevention and changing less desirable behaviours, rather than promoting healthy behaviours (Frerichs, Ataga, Corbie-Smith, & Tessler Lindau, 2016).

The ability to have control over one’s health is essential for empowerment, and adolescence is a critical time period. During adolescence, individuals develop their cognitive, economic, emotional, and social capacities that will influence how they think about their health and what influences their decisions and actions (Patton et al., 2016; World Health Organization, 2014). For example, adolescents’ cognitive abilities, such as their abstract thinking and reasoning, are developing (Patton et al., 2016). Similarly, their purchasing power often increases which can influence their health-related behaviours such as deciding what snacks and foods to buy, Thus, for adolescents, empowerment means both to experience a control over aspects of their environment that affect their health and to control themselves so that their actions lead them to choose healthy alternatives (Tengland, 2007). This can be promoted by interventions that provide information as well as involvement in different health-related activities to promote their self-confidence in new contexts. Encouraging adolescents to influence interventions is important for them to develop active learning strategies through hands-on activities (Greene, 2013). Conducting such activities can contribute to their autonomy as they learn about alternatives and choose to act on this new knowledge (Tengland, 2007).

This study was conducted as part of an evaluation of an empowerment-based school intervention, with a focus on healthy food and physical activity habits in an urban disadvantaged community. The aim of the study was to describe the adolescents’ experiences of empowerment from different perspectives. The following research questions were thus addressed:

1. What did the adolescents’ experience contribute to or impede their sense of participation in the intervention?
2. What intervention aspects did the adolescents’ experience that influenced their perceptions and behaviours related to healthy habits regarding food and physical activity?
3. How did their classroom teachers perceive the adolescents’ participation in the intervention (with a focus on the aspects mentioned in research questions 1 and 2 above).

## Methodology

To provide an opportunity for the participants to share their experiences, focus groups were conducted. Dahlin-Ivanoff and Hultberg (2006) argue that focus groups allow participants to relate to each other’s stories and shared realities, which in turn might stimulate them to share their experiences. Chen, Shek Daniel, and Bu (2011) argue that it is necessary to understand adolescents’ subjective interpretations of reality to understand the motivations for their behaviours. This study was thus based on a descriptive-interpretative perspective (Elliott & Timulak, 2005; Graneheim, Lindgren, & Lundman, 2017), as we aimed to describe different aspects of how the adolescents experienced participating in the intervention, as well as interpreting the reasons for these experiences.

### Setting and participants

The intervention school was selected because it was located in a disadvantaged urban community where there was a political ambition to work with health promotion among school pupils. One of the researchers was part of a health-promotion network in this community, and the principal and the teaching staff of the school expressed interest in a health-promotion project. All 7th graders in the school (n = 54, between 12 and 13 years old) were recruited at baseline in 2014. At the endpoint measurements in 2016, all adolescents who had taken part in the intervention and were still enrolled in the school were invited for the focus group interviews. Five pupils were not available due to no longer being enrolled in the current school or due to extensive absences. Thus, 29 girls and 20 boys participated in the present focus group interviews. We triangulated the data collection by also including the perceptions of the classroom teachers regarding their observatons of the adolescents’ experiences of participating in the intervention.

The school was situated in an area characterized by low SES, with a significantly lower average income, a higher degree of unemployment, and a higher multicultural composition of the population as compared with the municipality average (Gothenburg municipality, 2015). In this school, 49% of the pupils had passable grades in all subjects in ninth grade compared to 68% in the city as a whole. A majority (94%) of the pupils had a foreign background (i.e., were not born in Sweden or were born in Sweden but both parents were born elsewhere), compared to the municipality average of 34% (Swedish National Agency for Education, 2015a, 2015b). On a group level, individuals in the area also experienced poorer health (self-reported) and shorter life expectancy than the municipality average (Gothenburg municipality, 2017).

This intervention, called the “How-to-act?” project, were approved by the regional ethics review board in Gothenburg (#2014/469–14). All participants and their parents/legal guardians provided written and informed consent (in Swedish, Arabic or Somali) prior to the intervention. They also received information that participation was voluntary and that they could choose to leave the project at any time without having to disclose their reasons for doing so.

### The intervention

The three semester long intervention had a focus on healthy food and physical activity habits. The specific intervention components were developed and implemented through shared decision-making between the researchers and the participants. The intention was to enable empowerment through partnership with the adolescents (Nutbeam, 1998) by letting them express their perceived health-related needs, discussing how to access health information, and by facilitating skills development.

To support a sense of empowerment and facilitate participation throughout the intervention, health coaching was used as a continuous communication technique between the researchers and the adolescents. Health coaching aims to facilitate reflection, confidence in own ability, and identify strategies for health-promoting action (Olsen, 2014). The intention of having a health coaching approach was to support the adolescents in articulating their health-related ambitions, listen to their ideas about meeting those, and try to realize them by developing intervention activities.

Different groups were created with adolescents that shared similar health-related goals. The intention was to facilitate a feeling of connection and cooperation, and also to enable the researchers to tailor their support to the individual group’s goals and needs. To explore the adolescents’ goals and needs, individual interviews (n = 52) and focus group interviews (n = 10) were conducted during the first semester (Jonsson, Larsson, Berg, Korp, & Lindgren, 2017). Written statements of the adolescents’ individual goals with the intervention were also collected to supplement the interviews. Together, these insights formed the basis for the initial intervention activities.

The aim, type and content of each activity were thus guided partly by the participants’ expressed needs, and partly by common reasonable actions for implementation (e.g., that the content was possible to deliver within the given time frame and physical environment). The activities were carried out in collaboration with the classroom teachers, with members of the research group leading each health-promotion session.

Typical intervention activities included preparation of healthy vegetarian meals; searching, compiling, and presenting the benefits of a healthy diet to each other; meeting with the person responsible for school meals in the municipality; theme days with physical activities (e.g., playing sports); food-related workshops (e.g., identifying the amount of sugar in common foods); visiting a health exhibition; and classroom workshops concerning critical discussions around societal body-ideals.

To provide information to the participants and their parents/legal guardians, a website describing the project “How-to-Act?” was used. As 49 of the 54 adolescents in the intervention reported that they had a Facebook account, a specific group was created on this platform and administered by the researchers. Anonymous accounts were also created so that non-users could get an account of the intervention. A majority, 31, of the pupils joined the group. The Facebook group was utilized in a variety of ways during the intervention, with the overall aim being to facilitate participation and provide a forum for communication between the adolescents and the researchers. For example, participants were encouraged to post photographs during intervention activities, such as preparation of foods, while the researchers posted information and reminders on upcoming health-promotion sessions and provided feedback (comments and photographs) from previously implemented health-promotion sessions.

### Focus groups with the adolescents

As part of the endpoint measurements of the intervention, nine focus groups were conducted with the adolescents during the final semester over a period of two months in fall/winter 2016. Each focus group consisted of 4 to 7 pupils and was audio-recorded. The focus groups were conducted in the school and were constructed based on the groups that the adolescents most often worked within during the intervention. This made it more likely that they shared many experiences and that they knew each other well which has been shown to facilitate an environment of trust (Dahlin-Ivanoff & Hultberg, 2006). In line with recommendations by Dahlin-Ivanoff and Hultberg (2006) as well as suggested by the classroom teachers, boys and girls were interviewed separately with the exception of one mixed group. See Table I for an overview of the focus groups.

**Table I.** Overview of the focus group interviews with the adolescents.

Focus group interviews	Participants
1	6 girls
2	4 girls
3	6 girls
4	6 girls
5	5 girls
6	7 boys
7	5 boys
8	4 boys
9	2 girls and 4 boys

One senior researcher led the groups and a PhD candidate (CH) or researcher assisted by taking notes and managing the audio recordings. All of the researchers, three women (CB, CL, ECL) and one man (PK), that lead the focus groups were experienced in focus group methodology in their research areas of food and nutrition and sport science. To ensure trustworthiness of data, the two PhD students that were responsible to implement most of the ntervention activities, LJ and AF, did not participate during these focus group interviews.

Focus groups started by everyone introducing themselves. The leader then clarified the purpose of the focus groups. The questions focused on how the adolescents experienced the intervention with regard to positive or negative influences on empowering opportunities for learning. A large sheet of paper with “Participation” printed on it was displayed when we talked about the adolescents’ experiences. To make the concept of participation more tangible, we used the cue words “cooperate,” “deciding,” “feeling listened to,” “taking responsibility,” and “being able to influence.” Similarly, when we talked about learning, the printed word “Learning” was shown together with the cue words “food,” “physical activity,” “body,” and “health” to focus the learning experiences towards these areas. Cue words facilitating reflections with regard to learning were “being able to,” “daring to try,” and “new habits.”

Images (n = 23) from intervention activities were used to facilitate conversation and to help the adolescents remember the various intervention aspects. When the adolescents were asked what aspects had influenced them, they would select an image illustrating this aspect and talk about it in more detail. The participants could also select a blank sheet if they did not find an image that corresponded with what they wanted to talk about. They were encouraged to talk about both positive and less positive intervention experiences. If certain images were repeatedly not selected, the researcher asked the group why these were not selected. The same was done if the adolescents did not talk about using communication technology in the intervention. Questions were also asked to continuously engage the whole group when talking with one participant, such as: ‘Do any of you share this experience’ or “Did any others experience this in the same way?.”

### Triangulating focus group interview

The researchers conducting the focus group interviews had also participated in intervention activities and interacted with the adolescents. We thus wanted to probe if what the adolescents described during the focus group interviews was influenced by them trying to match our expectations. The focus groups with the adolescents were therefore triangulated by adding their classroom teachers’ perceptions of what they had observed of the adolescents’ experiences of participating in the intervention. The goal here was to see how similar the teachers’ observations were to what the adolescents described to us. The classroom teachers, three women and one man, were invited for a separate focus group interview. The classroom teachers had been part of numerous intervention activities, such as assisting during practical activities in school, and they had known most of the adolescents for several years.

This focus group was conducted at the university department. It was audio-recorded with three of the researchers present (CB, ECL, PK) and verbal consent having been obtained. This focus group was conducted a few weeks after the focus groups with the adolescents. We used the same images from the intervention activities to stimulate the discussion. The questions focused on what the teachers had heard the adolescents saying about the intervention in school, as well as the teachers’ own observations from participating together with the adolescents during intervention activities.

### Data analysis

An abductive analysis, using both inductive and deductive approaches, was performed to analyse the focus group materials by means of qualitative content analysis (Graneheim et al., 2017; Graneheim & Lundman, 2004). An initial inductive categorization was conducted to describe what the adolescents experienced as contributing to or impeding their participation in the intervention, as well as the intervention aspects that influenced their perceptions related to healthy food and physical activity habits. To interpret how these descriptions related to the adolescents’ empowerment and their involvement in the intervention, an overarching theme was generated. This descriptive theme reflected explicit statements of what the adolescents talked about in relation to the abilities Tengland (2007) argued are needed to gain control over one’s health, as well as Greene’s (2013) thoughts of intervention aspects that facilitate adolescents’ active involvement.

Based on the research questions, the analysis was performed in the following steps:

- The audio recordings from the focus groups were transcribed verbatim. These were listened to, and the transcripts read, in their entirety repeatedly, to understand the contexts and dynamics of the focus groups.
- Sentences or phrases containing features related to each other through their content and context, and of relevance to the research questions, were highlighted as meaning units. These meaning units were inputted to a table to assist the categorization process. The categorization process was thus transparent as it allowed the rest of the research group to follow the steps in the categorization, and it provided an overview of the different meaning units which allowed for comparison.
- As the meaning units consisted of several sentences and sometimes long paragraphs, they were shortened into condensed meaning units. Using an inductive approach, these meaning units were abstracted to codes. The codes functioned as labels and described the meaning units in relation to the research questions.
- Similar codes, reflecting the same aspects of the text in relation to the research questions, were collapsed into categories and subcategories. These categories were discussed and revised several times within the research group.
- When consensus was reached, we had generated 5 final categories and 11 subcategories. The categories captured the meaning of several subcategories, but they referred to a descriptive level of content, staying close to the adolescents’ descriptions. The categories expressed the manifest content of the text, aiming to answer the question “what?” (Graneheim & Lundman, 2004). The subcategories were internally homogeneous, as they consisted of codes that shared similar features. Externally, the subcategories were heterogeneous, as the same code could only be assigned to one subcategory.
- A descriptive theme that intersected with the categories was generated to describe the content on a latent level and forming a unifying red thread running through the categories (Graneheim et al., 2017).
- The categorization from the focus group with the teachers was analysed in a similar way, by identifying meaning units and creating codes inductively. These codes were thereafter connected and organized within the categories generated from the focus groups with the adolescents.

This analytical procedure enabled the experiences of individual adolescents to be visible, as the analysis focused on the manifest content by staying close to the transcribed text (Steps a-c). By creating categories and a theme, we were able to capture the adolescents’ voices as a collective (Steps d-f). The analysis is depicted as a linear procedure, but the actual analytical procedure was performed by going back and forth to continuously discuss and reflect critically upon the codes, categories, and theme. The analytical approach was to move from the text, the concrete and specific, towards a more abstract and conceptual understanding (Graneheim et al., 2017). The first author (CH) initiated and led the analysis together with the research group. The research group (co-authors) consisted of researchers and PhD students with backgrounds from different research fields, bringing nursing, nutritional, pedagogical, sociological, and psychological perspectives to food and physical activity. See Table II for an example of the categorization procedure.

**Table II.** Examples to illustrate the categorization procedure.

Meaning unit	Code	Subcategory	Category
We usually work more individually in school. So, it was very nice and fun to collaborate and work together.	Collaborating was fun as they normally work individually	Engaging doing things together	Interacting and cooperating with peers
It is fun to try to collaborate with people that one does not spend so much time together with normally. So that was a nice experience.	Collaborating with new peers was fun		
You might not feel so comfortable but you learn more when you work with those that you do not normally spend time with.	Learning more when working with less familiar peers	Learning from and with each other	
Everyone tried their best to help out and prepare meals together. So if you do not cook that much, you can learn from others who are more experienced.	Learning from more experienced peers		

## Findings

The adolescents felt that many of the intervention aspects that contributed to or impeded their sense of participation (research question 1) were similar to those aspects that they described as influencing their perceptions related to healthy habits (research question 2).

For the category *Opportunities to influence and have a dialogue*, the adolescents stated that by deciding and having the ability to influence the intervention they experienced involvement and also that they could learn from planning and by taking responsibility. For the categories *Interacting and cooperating with peers* and *Active learning in new activities*, the adolescents said that it was engaging to work with peers in active learning activities such as preparing meals and practicing sports. The adolescents also said that this could make them more aware of how to better follow healthy lifestyles such by preparing vegetable-based meals and also by working with more experienced peers. The category *Health information and instructions* concerned health information and instructions which the adolescents mentioned influenced their understanding of their health behaviours and how to conduct sports. For the category *Visualizing and reflecting over one’s practices*, the adolescents described that by using digital tools to visualize their own behaviours related to food and physical activity, the adolescents experienced that they could reflect and become aware by viewing their behaviours in new ways.

**Figure 1.**
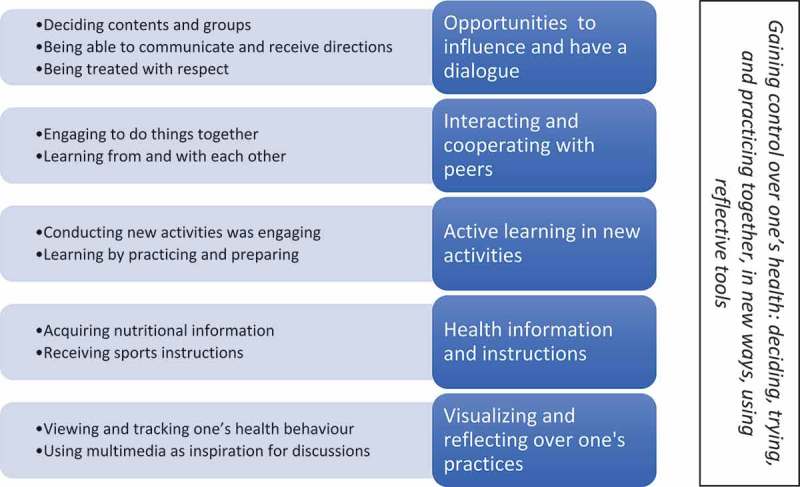
Illustration of subcategories, categories, and theme.

These intervention aspects mentioned by the adolescents influenced different abilities that are important for them to gain control over their health. The adolescents’ also mentioned which intervention aspects had influenced their involvement in these activities. An overarching theme was therefore generated intersecting with the categories: *Gaining control over one’s health: deciding, trying, and practicing together, in new ways, using reflective tools*. See Figure 1 for an overview of the subcategories, categories, and theme.

The five categories are illustrated below by quotes that are characteristic for each category, using fictitious names. When quotes are presented, “I” refers to the interviewers and “FG#” refers to the specific focus group interview. It is also indicated if the focus group consisted of girls or boys only, if it was mixed, or was with the teachers.

### Opportunities to influence and have a dialogue

The adolescents described feeling listened to and realizing that they could participate in the decisions about the intervention activities and the group configurations. They also said that it was fun and engaging to plan activities and events, as expressed by participant Anna: “I thought it was really nice that we got to decide on the groups and games, and in the food groups as well, so it was fun to be a part of this’ (FG3, girls). Often the groups could decide according to what the majority wanted which was appreciated. According to Nadia: ”Some got to decide the recipes, some got to decide on the groups, and some got to decide the activities. I liked it because then you could do what the majority wanted to do’ (FG3, girls).

However, the fact that the adolescents decided through majority decisions also left some participants with a sense of being forced to do what others had decided, which was illustrated by one participant. I: “What did you experience as less positive”? Bilan: “To do things that you didn’t want to do. For example, many wanted to play soccer and then there were some that did not want to play soccer.” I: “Did you have to play soccer then?” Bilan: “Yes” (FG1, girls).

The teachers mentioned that they observed that the adolescents appreciated having an opportunity to assume responsibility and that they noticed that they had been empowered from it. Minna: “They planned for weeks, ingredients, what to buy and everything. So they partook in that. Most of them were girls, and they were really happy. N and A were very excited over what they accomplished, having been in the kitchen, acting as leaders, planning, and were impressed” (FG, teachers).

The adolescents noted that information about the intervention in relation to influencing part of the project was positive. For example, the adolescents mentioned using the Facebook group as a positive experience because it allowed them to access information about activities: Kryztina:“It was actually really good. You get information.” Maryam: “Yeah, you get information, so you know in advance what to do, so you are more prepared and have more control over what goes on. That is really good” (FG5, girls). For example, the adolescents thought they were better prepared when they knew in advance how the activities would be carried out and that it was easier for them to participate in activities that required planning, such as bringing sports clothes. The adolescents also mentioned that it was positive that the researchers were part of the online group as it allowed them to ask questions, such as expressed by some boys: Osama: “Yeah, the group was good, cause when you had questions you could ask them in the group.” Reza: “Yeah, and you replied quickly, and when we had questions you answered them” (FG8, boys).

Also related to the adolescents’ experiences of participation was that the intention of the activity was negotiated and understood by everyone. Adolescents said that they sometimes could not clearly understand or see the point of an activity. An activity that was recurrently brought up in this regard was walking in the woods. The adolescents perceived that walking, without doing an activity, was pointless, and that they could not understand the goal with it or learn from it: Fawzia: “We walked and walked, but didn’t get anywhere, and then we went back again. I thought we would go somewhere.” Nadia: “Me too, and then we didn’t get anywhere” (FG3, girls). Related to such misunderstandings, the adolescents stressed the importance for them to decide on activities early during the intervention, as expressed by some boys. Damir: “That is important. You have to let us decide early on, so it will be right.” Talim: “Yeah, he is right, you have to let us decide in advance.” I: “How do you want to influence it?” Talim: “At the start of the semester ask us, ‘What do you want to do?’” (FG6, boys).

The adolescents appreciated being treated with respect and taken seriously. They mentioned an encounter when they were treated disrespectfully by a representative from the school meal services. The adolescents had prepared questions to ask the representative but found that the representative avoided answering their questions, that she displayed an arrogant attitude, and that she did not want to engage sincerely with participants.

Faduma: ‘She spoke with such a cold tone. I thought that was rude of her. When we asked her a question, she ignored it all the time. Instead she would turn to another pupil. So we repeated the question, but she didn’t even listen’ (FG3, girls).

Because the adolescents’ did not get a clear connection they did not find the discussion meaningful. This particular occasion was also echoed by the classroom teachers.

Carl: It was very difficult when the school meal representative was here. It was a very tense situation. The pupils can be provocative, and if you cannot handle that, then it will be problematic. It was constantly a feeling of ‘that is not how I would have responded to that’, simply put (FG, teachers).

### Interacting and cooperating with peers

The adolescents mentioned that they appreciated spending time together. They said that it was fun to get to know peers that they normally do not socialize with during the school day and to connect better with existing friends. This general engaging aspect of the intervention was expressed by two girls. Hibaaq: “I mean, if you look at the positive aspects, we have had a really nice time and spent time together.” Kadifa: “Yeah, we have got to know each other better too” (FG1, girls). Along the same lines, the adolescents also highlighted the Facebook group, as they said that it allowed them to interact with peers. Faduma: “Almost everyone is part of the group, so you can see who has seen, who have commented, so you can comment back and stuff like that, so that was good” (FG3, girls).

The adolescents described one aspect that contributed to the positive aspects of working with peers which was that that the collaboration worked out well and often exceeded their initial expectations. For example, the girls described that they were somewhat surprised that the boys showed responsibility and took activities so seriously.

Kryztina: I actually thought that this day would end in disaster and chaos, that no one would be able to prepare the food. But it was really nice. Everyone collaborated and no one did anything bad. Dinara: Exactly, we spent time together. I: Why do you think it turned out well? Kryztina: I thought that the boys would mess it up, disaster, run around. Dinara: They kind of did that. Kryztina: Yeah, maybe a little, but it was way better than what I expected it to be (FG5, girls).

In terms of learning, the participants explained that they had become better at collaborating with each other during activities, such as when playing soccer or basketball together and by preparing meals together. They also mentioned that to be able to focus on the activity and learn better, it was positive to collaborate with peers that they did not usually spend time together with.

Sabina: I don’t feel like I can focus when I am with my friends, and then I can’t learn anything from it, I will only remember what we have talked about. So when you are with people that you have not selected yourself, then you collaborate. Leyla: You don’t talk too much with each other. Sabina: Yeah, you don’t talk about other stuff . (FG2, girls).

Some were initially distressed when they found they were not working with their friends. However, as expressed by the teachers, they were usually often fine with it after the initial reaction. Minna: “They were complaining when they couldn’t be with their friends, but later they appreciated it” (FG, teachers).

Another element of working with peers was that the activities were experienced as engaging which could enable the adolescents to enjoy them more and lowered the barrier to start. Such an experience was expressed by one girl in relation to preparing meals. Leyla: “I couldn’t cook food and then when we did it together it was fun. It was fun after that because I was with S and Z. Then it was fun, and I started to prepare meals and stuff after that” (FG2, girls).

### Active learning in new activities

The adolescents expressed the feeling that it was fun to try various activities, some of which were new to them. For example, one boy mentioned that he overall experienced the intervention as positive because of this. Kaarim:“It was good, that we did many activities. It was a nice project since we could do so many activities” (FG7, boys).

Another aspect that was mentioned as positive was doing active things (e.g., sports) and practical things (e.g., preparing meals). The adolescents said that it was more engaging to conduct hands-on activities rather than listening and talking about health. Dinara: “I do not like to sit and listen about health; that’s boring.” Milena: “One should do more like this, food and stuff, like that” (pointing at an image of them preparing meals). Dinara: ‘Yeah, that’s right‘ (FG5, girls). The adolescents found it was engaging to do something practical as they did so much theoretical work in school already. Nina: “The most fun parts was when we did something practical, because one was writing during the whole school day, and then when we could move around, that was the best” (FG2, girls). The adolescents therefore suggested that assignments and writing activities should be limited in future interventions. Talim: “You should do less theoretical stuff, maybe just once per semester, and instead more practical activities, like more soccer and activities like that” (FG6, boys).

The classroom teachers also observed that the adolescents were less engaged during abstract activities compared with more practical activities. One teacher who participated both when the adolescents conducted written assignments and when preparing meals, noticed a difference between the two activities. Eifa:“It was boring for them to work with written activities, because they have so much to read in school. But they really enjoyed working in the home economics classroom and it was really nice and a good atmosphere there” (FG, teachers).

In terms of learning, the adolescents said that when they prepared meals, they could gain new skills and understand new things related to cooking food, such as noticing the number of recipes and realizing that they can prepare vegetarian food that tastes good. Participants also said that preparing meals made them understand healthy eating which inspired them to eat healthier. One participant mentioned that she realized that preparing tasty meals was easier than what she had previously imagined: Leyla:“I learned that one could prepare tasty meals by using simple things, such as vegetables and stuff, and that it doesn’t have to be fast food to taste good” (FG2, girls).

Similarly, the adolescents also mentioned that by trying physical activities, they could realize that it could be fun and not too difficult. They also believed that it made them try other types of physical activities, which was expressed by one girl. I: “What was it that made you start going to the gym?” Sabina: “I think it was the dancing. It has nothing to do with the gym, but I think it was when we were dancing because it was really fun” (FG2, girls). The teachers described similar experiences when the adolescents tried activities and realized that they could do it which could influence them outside the intervention context.

Nour: I also think that by doing these type of activities, some of the adolescents that didn’t know, or were not interested in these things, noticed that it could be fun. For example, a student who was nervous, we noticed that he really enjoyed preparing meals. So he has now enrolled in an individualized education programme, by practicing in a school canteen several times in the week. Minna: Wasn’t it you who were in his group when he prepared meals and observed how well he could organize the activity? Nour: Yes, he did very well, and I said to him, ‘You’re very good at this’, and he said that he really enjoyed preparing meals (FG, teachers).

### Health information and instructions

The adolescents stated that health information such as dietary recommendations, information about added sugar, and information about meal planning inspired them to eat healthier by consuming more fruits and vegetables. One girl mentioned that information about the recommended daily intake of fruit and vegetables motivated her:

Fawzia: Because we got this leaflet with information about how much fruit and vegetables one should eat, I found out that I did not consume enough, and then I got new habits by eating fruit and vegetables several times per day (FG3, girls).

Other participants mentioned that knowing more about added sugar inspired them to eat healthier food, as one girl said. Sana: ’I eat more fruits and vegetables.’ I: “What made you change?” Sana: “We learned more about what was healthy and not, how much sugar things contain” (FG4, girls). Similar experiences were described by the teachers. They believed that the adolescents were more knowledgeable about the health aspects of food after partaking in the intervention. Nour: “During biology, we have talked about food and health, and they knew a lot about it. About fibre and such, they have really learned a lot during these sessions when they talked about these things” (FG, teachers). Another teacher observed that the adolescents had become aware of health aspects on a more general level after the intervention.

Carl: During a social science class we discussed why entrance to bath houses is subsidized in Sweden so that children have free access to them. The first thing a lot of them said was because physical activity is important for health and that exercises like swimming are good for the body, instead of mentioning that because it is mandatory for Swedish students to be able to swim 200 meters. They had some really good arguments and reasoning around those health aspects, so I think they are more aware of health aspects in general (FG, teachers).

The adolescents also mentioned that they acquired knowledge and kills facilitating physical activities by being instructed in exercises and sports. One boy said that he learned more about how and why to stretch in sports, and another boy learned more about new martial arts after meeting an instructor. Raafid: “I got to learn MMA [mixed marital arts] and Taekwondo.” I: ‘What did you experience that was educational? Raafid: “The guy, he was an instructor” (FG9, mixed genders).

### Visualizing and reflecting over one’s practices

The adolescents described that seeing how much one had walked when using a pedometer and accelerometer was fun and that it could motivate one to walk more and start working out, as expressed by one boy. Reza:“Then you got like, how much should one walk during a week, and it was fun to see how much one had walked” (FG8, boys). Similarly, one girl said that self-reflection was motivating. Nadia: “When you see how much you have walked, you think, oh, is that how much or little I walk. You might get inspiration to walk more, or to start working out” (FG3, girls). Adolescents also reflected on their eating habits. At one stage they took photos of what they consumed for a couple of days, and they reported that they became more aware of what they ate. Some reported that the photos enabled them to reflect on their consumption, and some said that they started to modify their dietary habits as they became more aware what they were eating. Kadifa: “I was shocked that I ate so much sweets, but I feel like, that after that experience, I have tried to decrease my intake of sweets” (FG1, girls).

The adolescents also described becoming interested when discussing how beauty and body ideals are influenced by media. They mentioned that watching a video illustrating how images of models are manipulated and Photoshopped made them understand why it was important to be media critical: Alexandra: “Models and stuff, the internet can be Photoshopped, so you shouldn’t be affected by it, because you don’t always know what is real” (FG4, girls). A similar sentiment was also expressed by some boys regarding watching the same video: Damir: “It taught me to be source-critical, to not trust everything I see, to think before, that is what I learned” (FG6, boys).

The adolescents also talked about food and nutrition workshops as influential. The workshops utilized video clips to display health facts about energy drinks, and another component was the use of sugar cubes to illustrate added sugar in common food and beverages.

Faduma: You do not think about it when you eat it, but then, when you see it, you think, oh my God. Meri: You think, oh shit. Faduma: You think, oh shit, so much sugar. Meri: Shocking. Faduma: Things you thought were healthy are not healthy, like yoghurt (FG3, girls).

The adolescents also discovered that using interactive devices such as iPads to look for recipes and food information during workshops was preferable to using textbooks, because it provided access to more information and was easier to use. They also mentioned that using video and film clips during activities could make information easier to understand and suggested this for future interventions. Khaled: “You should include more activities that involve video.” I: “Why should we do that?” Khaled: “Because it is easier to understand using video” (FG7, boys).

## Discussion

The adolescents in this study highlighted several intervention aspects as influencing various abilities that are important for them to gain control over their health. Interestingly, many of these aspects influenced both their food and physical activity habits. This is positive as it is favourable to target both behaviours in health-promotion activities (World Health Organization, 2009, 2013).

The adolescents mentioned aspects such as being aware of healthy food options and how to conduct new physical activities. This awareness raising boosted their self-confidence to pursue healthy lifestyles and recognize healthy alternatives, which are important for empowerment (Tengland, 2007). The results also emphasize the importance of the adolescents’ engagement in their health and in the intervention. As such, these findings show similarities with key concepts in the theory of active involvement (Greene, 2013). This evolving theory is grounded in experiential learning principles and is based on social cognitive theory, and it is proposed that such activities stimulate reflective thought and action (Bandura, 1986; Greene, 2013).

### Active learning and group activities

The adolescents in this study identified preparing meals and conducting physical activities as both engaging and enhancing their learning. They found that when they prepared meals, they realized the number of recipes that existed, that plant-based meals could be tasty and that there were feasible, tasty, and healthy alternatives to fast food. These findings are in line with a study that evaluated the acceptability of eating interventions among European adolescents (Stok et al., 2016). The researchers found that adolescents prefer strategies that promote healthy eating, such as showing healthy alternatives, to those that discourage unhealthy eating. Also, in terms of physical activities, studies have highlighted that it is important to provide opportunities for adolescents to try new activities as it could overcome barriers to engaging in sport activities (Corder, Schiff, Kesten, & Van Sluijs, 2015). By trying these activities in the intervention, the adolescents mentioned that it made them try other activities outside the intervention, such as going to the gym or preparing more healthy meals at home. This is an important part of empowerment as self-confidence and belief in one’s own abilities indicate that people are more likely to take control over their own health-related situation (Tengland, 2007).

Adolescents and teachers alike expressed the belief that theoretical-based activities were less stimulating. These findings are in line with Greene’s (2013), who argues that it can be difficult to capture adolescents’ attention if the activities occur in a school setting or is perceived as similar to the normal school content. However, we found ways to work within the school setting by, for example, rearranging the classrooms to conduct active sessions.

The adolescents told us that working together with peers in school was more engaging than working alone. This finding tallies with socially situated experiential learning which holds that the social context is crucial for engagement because adolescents are generally more engaged with peers than they are with adults (Greene, 2013; Lederman & Stewart, 1998). Spending time with peers represents a valuable social contact that contributes to adolescents’ well-being. Adolescents who participate in social networks are generally found to have better perceived health and take part in more healthy behaviour as it helps them develop social skills, raise their self-esteem and establish autonomy (World Health Organization, 2016).

The adolescents described that trying new activities and acquiring new health information was exciting. They also found benefits from visiting new places such as the university department where the researchers worked to prepare meals together, or to visit museum exhibitions about health. Breinbauer (2005) and Greene (2013) argue that novelty is an important stimulant that can engage adolescents in health-promotion interventions. In terms of trying new activities, the adolescents also emphasized that sport instruction was valuable, which might be necessary if the activity is new. Other studies have cited the interest of adolescents in using mentors to instruct them in physical activities (Corder et al., 2015). Finally, it is important to consider the group dynamic. Several of the groups in this intervention were girls-only and some chose to dance together. Pearson, Braithwaite, and Biddle (2015) observed that girls-only settings facilitated girls’ physical activity and enabled them to move around more freely without feeling that they had to perform under the gaze of boys.

### Active involvement and information

The adolescents mentioned how much they appreciated having the opportunity to influence the intervention activities. They said that having a choice made them take responsibility, and the classroom teachers mentioned how proud the adolescents were of organizing a full day of activities, for example. Greene (2013) has also argued that adolescents will feel involved and excited about the intervention if they are included early in discussions. The adolescents in our study said that there could have been misunderstandings if the researchers had not listened to them first in discussing, for example, the goals of the activities. Allowing the adolescents a voice in decisions is also important from an empowerment perspective. As all relationships involving health have a power aspect (Larsson & Jormfeldt, 2017), it is important that the “experts” hand over some of the power to the participants to provide a joint approach (Tengland, 2007).

To enable the adolescents a degree of influence, it is also important to provide access to all relevant information, which is an important part of empowerment. The adolescents mentioned that the use of the Facebook group page enabled the adolescents to access information about intervention activities in advance and also to share information with peers. These findings support the work of Norman (2012), who argues that communication methods familiar among adolescents, and social media technology can enable adolescents to share health information in ways that fit their preferences. Our adolescents also explained that they appreciated using the Facebook group to communicate with each other and the researchers. However, when the adolescents were enrolled in grade 7, they said that they wished to engage less with digital technology as it undermined their health habits (blinded for peer review). Studies suggest that adolescents in general experience a duality of both positive and negative health influences when engaging with digital technology (Favotto, Michaelson, & Davison, 2017). However, similar to our findings, Favotto et al. (2017) noted that while adolescents might want to decrease the time spent with digital technology, they enjoy it for connecting with peers. This is an important area to explore further as reviews show that social media are increasingly used within complex health-promotion interventions targeting adolescents (Hamm et al., 2014). Using established social media platforms might be beneficial in removing physical access barriers of significance to health equity, particularly in at-risk, disadvantaged populations (Welch, Petkovic, Pardo, Rader, & Tugwell, 2016).

The adolescents mentioned that visualizing their health behaviours such as food consumption through photo-food diaries, made them more aware of what they were consuming. Studies indicate that mobile phone use enhances self-monitoring due to their ubiquity among adolescents and that they provide reasonable estimations of food intake (Foster et al., 2006). Smartphones might also be feasible to use among adolescents who are experiencing language difficulties, such as those with an immigrant background, and the use of photo-food diaries has also been successful when exploring healthy eating among ethnic minority students (Rodgers et al., 2016). Similarly, our adolescents stated that using pedometers made them reflect on their physical behaviours in new ways. Such findings have been noted among adolescents concerning how step data encourage adolescents to reflect on physical activity in everyday life (Bruselius-Jensen, Danielsen, & Viller-Hansen, 2014). These visual tools might enable the adolescents to view their health behaviours differently, providing them with greater empowerment in decision-making processes related to their health.

### Strengths and limitations

Conducting research interviews with adolescents is accompanied by power relations (Vähäsantanen & Saarinen, 2013). As two adults were present during each focus group, a power imbalance may have existed. The power imbalance may have constrained the adolescents’ opportunity to express themselves freely. It may also have contributed to the adolescents feeling inclined to answer the questions in a certain way such as answering in a socially desirable way. However, as Cyr (2016) argues, groupthink and desirability bias might provide focus groups with external validity. Cyr illustrates that while the final outcomes that emerge from a question in a focus group may not necessarily reflect every participant’s individual perception, the norms to conform reflect conventional conversations because social pressures permeate everyday social interactions.

The adolescents’ differing abilities to express themselves verbally and their willingness to do so was observed both within and between the focus groups. For example, some of the boys did not take the discussions seriously and interrupted the dynamic by being loud or by going in and out of the room. This probably hindered boys in the same focus groups to share their experiences, which likely led to less rich data generation from these focus groups. We also observed that the girls were in general more articulate than the boys, particularly the girls in the first three focus groups. This might relate to adolescent girls’ emotional and cognitive maturity, social factors such as gender norms, or their overall eagerness to participate in the intervention (Koolschijn & Crone, 2013; Wiium, Breivik, & Wold, 2015). However, this study’s results and categorization were based on all the focus groups. Meaning units from all the focus groups were represented in the categories and quotes from all the focus groups were included to illustrate the categories.

Using images of intervention activities as well as the sheets with preconceived cue words during the focus groups might have steered the adolescents to talk about certain aspects of the intervention. However, we believe that the use of images to facilitate reflection on intervention activities facilitated the focus groups. This was also described by the classroom teachers during their focus group, when they said that it would have been difficult to remember all the activities that made up the two-year intervention without using these images. Also, 23 images were used, as we tried to represent the range of activities that were central to the intervention.

One way to deal with issues of trustworthiness (Graneheim et al., 2017) was to include several researchers in the analysis. This is important for dependability as researchers might make different inferences and it is essential to address alternative interpretations. Another aspect of trustworthiness is credibility, that is, selecting participants who have experienced the phenomenon under study. All adolescent intervention participants were invited to participate in the focus groups. Five pupils were not available during the time of their focus group and these adolescents might have contributed to conflicting or complementary perspectives not addressed in this study. Generally, the study participants were a heterogeneous group, which should be considered a strength as it pertains to the transferability of study findings to other settings. The adolescents’ represented a broad array of backgrounds and some were born in Sweden, though many were born in countries on different continents.

The researchers conducting the focus groups and analysing the data were also involved in conducting some of the intervention activities. There might thus be a risk of bias towards the interpretation of the adolescents’ experiences. Therefore, to increase credibility we included the classroom teachers’ observations of the adolescents’ experiences of participating in the intervention. The teachers had known the adolescents for several years and were included from the start of the intervention. The teachers’ observations were similar to what the adolescents expressed during the focus group interviews with us. This might indicate that the adolescents did not shape their answers to conform with what they believed we wanted to hear. Also, during data analysis, we were careful to also consider negative aspects of the intervention as expressed by the adolescents.

## Conclusions

Based on the adolescents’ experiences and from how this intervention was developed and implemented by means of shared decision-making and cooperation, the findings contribute to understanding the importance of the different empowering aspects. Aspects such as novelty as a way to engage the adolescents, active learning, and early identification of ways to communicate with the adolescents to suit their preferences. The result also highlights the importance that everybody in empowerment-based interventions adapt to an approach of openness and tolerance. Before initiating interventions, researchers might be advised to establish a code of conduct to include not only the responsible researchers but also everyone else who is involved.

Generally, the intervention aspects highlighted by the adolescents in this study do not demand significant additional resources and are affordable and attainable as they mostly utilized existing facilities. It is therefore reasonable to believe that they could serve as inspiration for future interventions in empowerment-based health promotion projects in low-SES areas.
